# Preconcentration of Trace Neonicotinoid Insecticide Residues Using Vortex-Assisted Dispersive Micro Solid-Phase Extraction with Montmorillonite as an Efficient Sorbent

**DOI:** 10.3390/molecules23040883

**Published:** 2018-04-11

**Authors:** Khwankaew Moyakao, Yanawath Santaladchaiyakit, Supalax Srijaranai, Jitlada Vichapong

**Affiliations:** 1 Creative Chemistry and Innovation Research Unit, Department of Chemistry and Center of Excellence for Innovation in Chemistry, Faculty of Science, Mahasarakham University, Maha Sarakham 44150, Thailand; mai.2535@hotmail.com (K.M); jitlada.v@msu.ac.th (J.V); 2 Department of Chemistry, Faculty of Engineering, Rajamangala University of Technology Isan, Khon Kaen Campus, Khon Kaen 40000, Thailand; yanawath.sa@rmuti.ac.th (Y.S); 3 Materials Chemistry Research Center, Department of Chemistry and Center of Excellence for Innovation in Chemistry, Faculty of Science, Khon Kaen University, Khon Kaen 40002, Thailand; supalax@kku.ac.th (S.S)

**Keywords:** montmorillonite, dispersive micro-solid phase extraction, neonicotinoids, natural surface water and fruit juice samples

## Abstract

In this work, we investigated montmorillonite for adsorption of neonicotinoid insecticides in vortex-assisted dispersive micro-solid phase extraction (VA-d-μ-SPE). High-performance liquid chromatography with photodiode array detection was used for quantification and determination of neonicotinoid insecticide residues, including thiamethoxam, clothianidin, imidacloprid, acetamiprid, and thiacloprid. In this method, the solid sorbent was dispersed into the aqueous sample solution and vortex agitation was performed to accelerate the extraction process. Finally, the solution was filtered from the solid sorbent with a membrane filter. The parameters affecting the extraction efficiency of the proposed method were optimized, such as amount of sorbent, sample volume, salt addition, type and volume of extraction solvent, and vortex time. The adsorbing results show that montmorillonite could be reused at least 4 times and be used as an effective adsorbent for rapid extraction/preconcentration of neonicotinoid insecticide residues. Under optimum conditions, linear dynamic ranges were achieved between 0.5 and 1000 ng mL^−1^ with a correlation of determination (*R^2^*) greater than 0.99. Limit of detection (LOD) ranged from 0.005 to 0.065 ng mL^−1^, while limit of quantification (LOQ) ranged from 0.008 to 0.263 ng mL^−1^. The enrichment factor (EF) ranged from 8 to 176-fold. The results demonstrated that the proposed method not only provided a more simple and sensitive method, but also can be used as a powerful alternative method for the simultaneous determination of insecticide residues in natural surface water and fruit juice samples.

## 1. Introduction

Neonicotinoids are a relatively new generation of pesticides that have been introduced to the market since the launch of pyrethroids [1]. This group of insecticides includes nitro-substituted (dinotefuran, nitenpyram, thiamethoxam, imidacloprid, and clothianidin) and cyano-substituted (acetamiprid and thiacloprid) compounds [2]. They are most commonly used on rice, maize, sunflowers, rapeseed, potatoes, sugar beets, vegetables, and fruits crops [3]. Neonicotinoid insecticides act as agonists to insect nicotinic acetylcholine receptors (nAChRs), which play an important role in synaptic transmission in the central nervous system [1]. The widespread use of neonicotinoid insecticides at various stages of agricultural cultivation and during postharvest storage could give rise to serious risks for the health and safety of the consumers [4]. Consequently, restrictions in their agriculture use and maximum residue limits (MRLs) in some food commodities have been established [5]. The MRLs for neonicotinoids in fruit, vegetables, and cereals range from 0.1 and 1.0 mg kg^−1^ [6]. Thus, a sensitive and selective method for monitoring of neonicotinoid residues at low concentration levels is required to ensure food quality and to protect the consumer.

High performance liquid chromatography (HPLC) coupled with various detection systems, including ultraviolet [7], diode array [8], fluorescence [9], and mass spectrometry [10,11], is a preferred choice for neonicotinoid pesticide analysis [12]. Liquid chromatography coupled to mass spectrometry (LC-MS) has been demonstrated as an excellent method that enables analysis of neonicotinoid insecticides in real samples [13]. However, gas chromatography (GC) has also been reported for neonicotinoid determination [14] but requires some special conditions to reduce thermal decomposition. Capillary electrophoresis (CE) has also become an attractive approach for the separation of pesticide residues, but suffers from low sensitivity because of the short optical length of the capillary [15]. Generally, neonicotinoids appear in agricultural products at very low concentrations and in complex matrices, so sample preparation is a necessary step before analysis. Sample clean-up techniques are the most commonly employed, which comprise liquid-liquid extraction (LLE) [16], solid-phase extraction (SPE) [17], solid-phase microextraction (SPME) [18], and liquid-liquid microextraction (LLME) [19]. However, LLE suffers from the disadvantage of requiring large amounts of both samples and toxic organic solvents. On the other hand, SPE techniques typically require reduced amounts of organic solvents relative to LLE, but SPE sometimes suffers from analyte breakthrough when large sample volumes are analysed [6]. As a result, two types of microextraction methods have been developed, namely solid phase microextraction (SPME) [20] and liquid phase microextraction (LPME) [21]. LPME is based on the use of very low volumes (at the microliter level) of solvent and has its origin in the use of a drop of extraction solvent [22]. SPME integrates sampling, extraction, concentration, and sample introduction into a single solvent-free step. Although SPME and LPME eliminate and/or reduce the volume of consumed organic solvents, they are usually time-consuming processes [23]. In 2003, Anastassiades et al. reported a new approach for sample preparation called dispersive solid phase extraction (DSPE) [24]. In this method, the solid sorbent is added directly to a sample solution without conditioning, so the clean-up procedure requires only shaking or vortex and centrifugation. However, DSPE cannot provide enough cleanup for some complex matrices. More recently, a novel method called dispersive micro-solid phase extraction (d-µ-SPE) has been developed [24]. This technique is based on SPE methodology but uses a small amount of a solid sorbent (μg or mg range) dispersed in the sample solution to extract the target analytes. Compared to conventional DSPE, d-µ-SPE has the following advantages: simpler operation, less solvent consumption, and shorter time requirement. In d-μ-SPE, the natural and physicochemical properties of the solid sorbent are very important in order to achieve an accurate, sensitive, and selective determination of the target analyte. A variety of types of adsorbent materials have been used, including carbon nanotubes, graphene, inorganic nanoparticles, and polymer-based sorbents [25]. Clay has been investigated as a sorbent material due to its unique polarity, pore-size distribution, and high surface area [26]. There are three classes of clay, including illite, kaonite, and montmorillonite. Montmorillonite (MMT) is a class of natural clay that possesses a large surface area and high cation-exchange capacity. It has been demonstrated to serve as an effective sorbent [27]. In recent years, MMT has been successfully used as an adsorbent for extraction techniques, and therefore has potential in the adsorption of environmental pollutants [28,29,30]. MMT use has been reported for SPME [30]. MMT provides a high specific surface area due to its non-smooth, porous structure, resulting in higher loading capacity and thermal stability. However, it is very difficult to manufacture each stand with exactly the same coating thickness [31].

The aim of this work is to develop a simple d-µ-SPE for preconcentration of neonicotinoid insecticides in surface water and fruit juice samples combined with HPLC for determination of target analytes. In this research, MMT clay will be used as an efficient adsorbent in the d-µ-SPE of trace neonicotinoids. Vortex agitation was used to accelerate the extraction efficiency of the proposed method. Various experimental parameters which effected the extraction efficiency were evaluated. Application of the developed method is proposed for extraction and preconcentration of neonicotinoid residues in natural surface water and fruit juice samples prior to HPLC analysis.

## 2. Result and Discussion

### 2.1. Optimization of the VA-d-µ-SPE Procedure

The main factors affecting the extraction efficiency of the vortex-assisted dispersive micro-solid phase extraction (VA-d-µ-SPE) procedure, including amount of sorbent, sample volume, salt addition, type and volume of extraction solvent, vortex time, and the centrifuge extraction time, were optimized. The extraction efficiency in terms of peak area was used as the experimental response. A one-at-a time procedure was followed to understand the individual influence of each parameter. The optimization was carried out on an aqueous solution containing 500 ng mL^−1^ of each target analyte. All the experiments were performed in triplicate, and the means of the results were used for optimization.

#### 2.1.1. Effect of the Sorbent Amount

**Figure 1:**
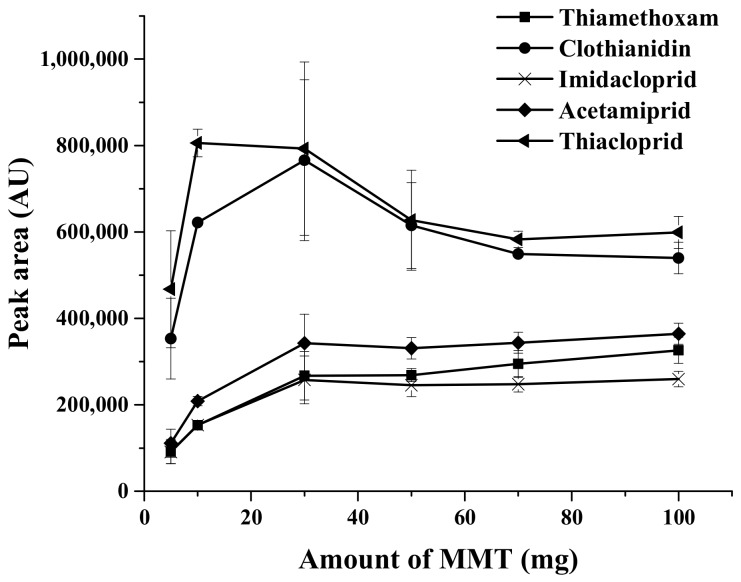
Effect of amount of montmorillonite (MMT) sorbent on the extraction efficiency, using a sample volume of 10 mL, 0.1 g of Na_2_SO_4_, and 200 μL of acetonitrile as desorption solvent. AU: Absorbance units.

The amount of sorbent has an important effect on the extraction efficiency of the proposed extraction method. In the present work, the sorbent was directly added into the sample, and dispersed with the support of vortex agitation [32] during the extraction process. Different amounts of MMT Cloisite 10A clay sorbent in the range of 5–100 mg were studied, while the other parameters were kept constant. As can be seen in Figure 1, the extraction efficiency in terms of peak area increased as the amount of MMT sorbent increased up to 30 mg. This may be due to the number of active sites and high surface area of MMT that increase the extraction efficiency. Efficiency then decreased for values higher than 30 mg. The reason may be that excessively strong adsorption leads to difficulty during the desorption process. With higher amounts of MMT, the extraction efficiency did not present a marked enhancement. Therefore, 30 mg of MMT sorbent was sufficient for effective extraction and was used for further experiments. 

#### 2.1.2. Effect of Sample Volume

**Figure 2:**
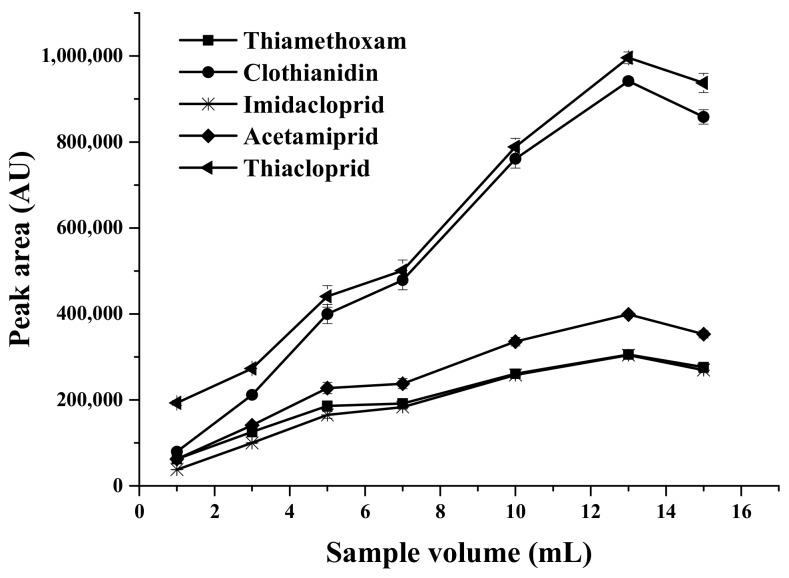
Effect of sample volume on the extraction efficiency, using 30 mg MMT sorbent, 0.1 g of Na_2_SO_4_, and 200 μL of acetonitrile as desorption solvent.

Sample volume is an important parameter which influences the extraction efficiency. An increase in the ratio of volume of aqueous phase to adsorbent leads to a significant increase in the extraction efficiency. On the other hand, an increase in the sample volume may result in a decrease in extraction efficiency in a given time. The effect of sample volume on the extraction efficiency was studied in the range of 1–15 mL. As shown in Figure 2, the highest peak areas were obtained using a sample volume of 13 mL. Therefore, 13 mL of sample volume was selected for further study.

#### 2.1.3. Ionic Strength

**Figure 3:**
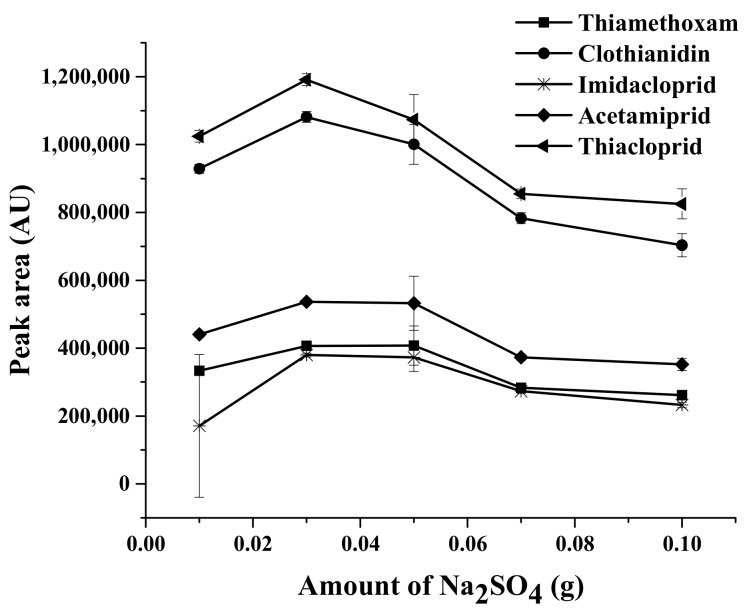
Effect of amount of Na_2_SO_4_ on the extraction efficiency, using 30 mg MMT sorbent, sample volume of 13 mL, and 200 μL of acetonitrile as desorption solvent.

Salt addition plays various roles in the microextraction processes, depending on the nature of the analyte and sorbent in terms of hydrophobicity and hydrophilicity and their interaction [33]. It can reduce the solubility of the target analytes and either reduce the solubility of the organic solvent in water or reinforce partitioning of the target analytes into the organic phase [34]. The influence of ionic strength was studied by the addition of several salts. Various kinds of salts (e.g., NaNO_2_, NaCl, Na_2_SO_4_, CH_3_COONa, and Na_2_CO_3_) were examined with the amount of each salt being kept constant at 0.1 g and the results were compared with that obtained from the process without salt addition (data not shown). It was found that Na_2_SO_4_ provided higher extraction efficiency in terms of peak area of neonicotinoids and gave better chromatograms than other salts. Therefore, Na_2_SO_4_ was selected for the further studies. On the other hand, with the increase of salt concentration and ionic strength, the salting-in effect can be a dominant phenomenon [35] whereby polar molecules may take part in electrostatic interactions with the salt ions in solution; thus, the mass transfer is diminished [36]. To probe the effect of salinity on extraction performance, experiments were accomplished by adding different amounts of Na_2_SO_4_ in the range of 0.01–0.5 g. As shown in Figure 3, the peak area increased to a maximum when the amount of Na_2_SO_4_ was increased to 0.03 g, and then decreased with greater Na_2_SO_4_ amounts. Therefore, 0.03 g Na_2_SO_4_ was used in this study. 

#### 2.1.4. Effect of Type and Volume of the Desorption Solvent

**Figure 4:**
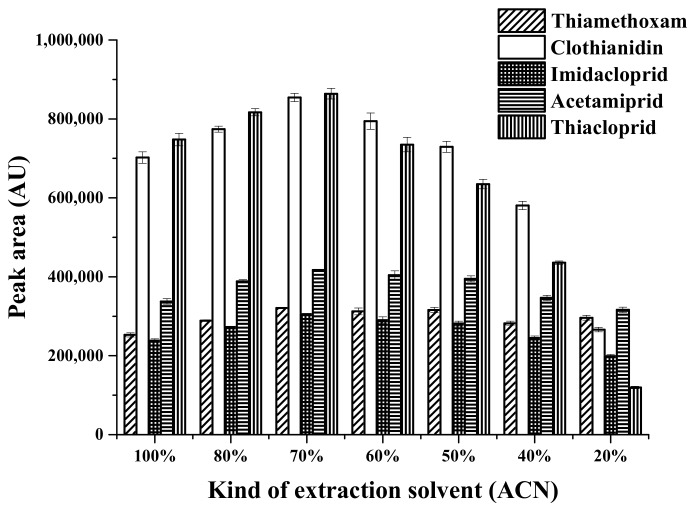
Effect of extraction solvent on the extraction efficiency, using 30 mg MMT sorbent, sample volume of 13 mL, and 0.03 g Na_2_SO_4_. ACN: Acetonitrile.

The organic solvents applied to elute the target analytes observably affected the degree of desorption of compounds from the adsorbent. According to the principle of like dissolves like, polar solvents are useful for dissolution of polar analytes. Based on this principle, polar desorption solvents were tested for desorption of neonicotinoid insecticides. In this study, 20–100% (*v*/*v*) acetonitrile was studied. As can be seen in Figure 4, the highest extraction recovery for neonicotinoids was obtained with 70% (*v*/*v*) acetonitrile as the desorption solvent. Therefore, 70% (*v*/*v*) acetonitrile was chosen as the desorption solvent for the following experiments. 

**Figure 5:**
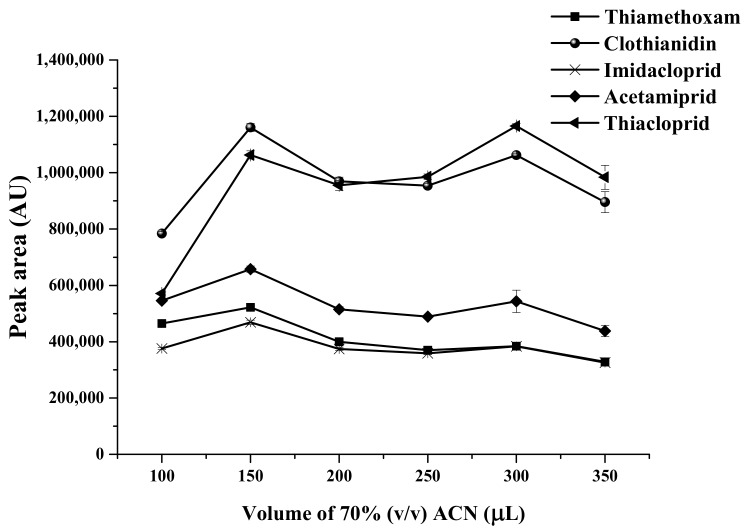
Effect of volume of 70% acetonitrile on extraction efficiency using 30 mg MMT sorbent, sample volume of 13 mL, and 0.03 g Na_2_SO_4_.

The volume of the desorption solvent (70% (*v*/*v*) acetonitrile) was another significant parameter that closely effected the extraction efficiency. Different volumes of desorption solvent ranging from 100 to 250 µL were investigated. As can be seen from Figure 5, the highest extraction efficiency in term of peak areas of the target analytes was obtained with 150 µL of desorption solvent. This reason could be ascribed to the fact that the force between target neonicotinoids and sorbent was strong and the analytes needed more desorption solvent for complete elution. When the volume of desorption solvent more than 150 µL, the peak area of the target analytes decreased due to the dilution effect. Therefore, 150 µL of 70% (*v*/*v*) acetonitrile was selected for use in the following experiments. 

#### 2.1.5. Effect of Vortex Time

Vortex agitation was selected for acceleration of the extraction process to enhance the extraction efficiency as it provided vigorous stirring of sample and the sorbent [37]. The effect of vortex time was investigated in the range of 30–240 s (data not shown). It was found that equilibrium was achieved received within 60 s of loading time and 120 s of eluting time. Beyond this point, the extraction efficiency decreased. Therefore, 60 s of loading time and 120 s of eluting time was used for the VA-d-µ-SPE process. 

#### 2.1.6. Reusability of Adsorbent

The reusability of the MMT sorbent for extraction of neonicotinoid insecticides was studied. In order to ensure elimination of residues on the MMT before extraction using the proposed method, the adsorbent was washed with 5 mL of methanol. It was found that the extraction efficiency in terms of peak area decreased after 4 cycles (data not shown). This indicates that MMT possesses excellent reusability as an efficient adsorbent.

### 2.2. Analytical Performance of the Proposed Method

To study the analytical performance of the proposed method, the analytical parameters, including linear ranges, correlation coefficients (*R^2^*), precision, limit of detection (LOD, S/N = 3), limit of quantification (LOQ, S/N = 10), and enrichment factors (EFs), were investigated under the selected conditions. The experimental results are summarized in Table 1.

**Figure 6:**
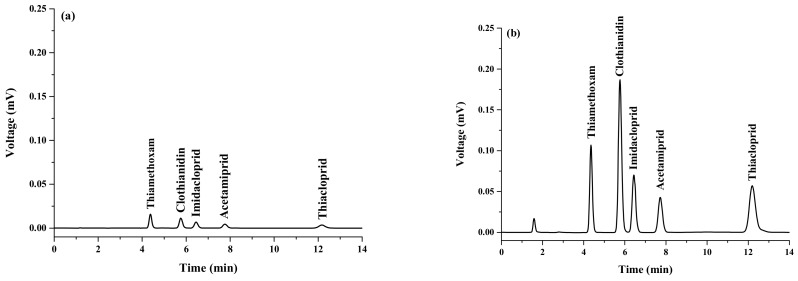
Chromatograms of standard neonicotinoids obtained (**a**) without preconcentration, and (**b**) with preconcentration using the proposed method (concentration of all standards was 500 ng mL^−1^).

All analytes exhibited good linearity in the range of 0.5–1000 ng mL^−1^ with a correlation of determination (*R^2^*) greater than 0.99. LOD and LOQ were evaluated by the analyte concentration giving the signal to noise ratios (S/N) of 3 and 10, respectively. LODs of the studied analytes ranged from 0.005 to 0.065 ng mL^−1^, while LOQs ranged from 0.008 to 0.263 ng mL^−1^. To test the reproducibility of the proposed method, intra-day and inter-day precision was studied by replicate injection of the standard mixture of 50 ng mL^−1^ on one day (*n* = 3) and several days (*n* = 3 × 3). Good precision was obtained with relative standard deviations (RSDs) less than 0.44% for retention time and 7.17% for peak area. The enrichment factor (EF), defined as the concentration ratio of the analytes in the settled phase (Cset) and in the aqueous sample (Co.), ranged from 8 to 176-fold. Chromatograms of the studied neonicotinoid pesticides obtained from direct HPLC and preconcentrated by the proposed VA-d-µ-SPE are shown in Figure 6. 

### 2.3. Real Samples Analysis

The proposed µ-SPE method was utilized for the simultaneous determination of neonicotinoid insecticides in natural and fruit juice samples from local markets and supermarkets in Maha Sarakham (Thailand) province. The results are summarized in Table 2. It was found that no residue of the studied neonicotinoids was observed in the natural surface water (data not shown) and longan samples. For the watermelon and grape samples studied, all the studied neonicotinoid insecticides were detected in the range of 0.005–0.27 ng mL^−1^. However, the amounts of neonicotinoid pesticides found in the fruit samples were lower than the maximum residue limits (MRLs) established by the EU (acetamiprid, 0.5 mg kg^−1^ in grape; imidacloprid, in grape; clothianidin, 0.9 mg kg^−1^ in grape).

**Figure 7:**
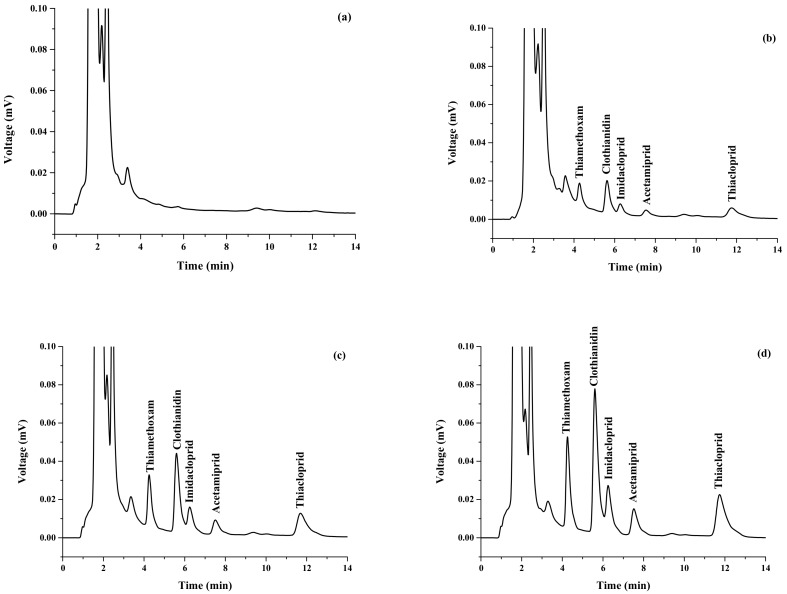
Chromatograms of (**a**) longan sample, (**b**) longan sample spiked with 100 ng mL^−1^ of each neonicotinoid, (**c**) longan sample spiked with 250 ng mL^−1^ of each neonicotinoid, and (**d**) longan sample spiked with 500 ng mL^−1^ of each neonicotinoid.

In order to validate the accuracy of the established method, the fruit juice samples were spiked with neonicotinoid insecticides at concentration levels of 250 and 500 ng mL^−1^ (data not shown). For all the target analytes in the spiked samples, the recoveries and RSDs were obtained in the range of 70–138% and 0.1–7.5%, respectively. Figure 7 shows typical chromatograms of a longan sample extracted by solvent extraction followed by VA-d-µ-SPE.

### 2.4. Comparison of the Proposed VA-d-μ-SPE Method with Other Sample Preparation Methods

The proposed VA-d-μ-SPE was compared to other sample preparation methods for analysis of neonicotinoid insecticide residues. As summarized in Table 3, the analytical performance of the proposed method was comparable and, in some cases, had distinct advantages over other reported studies. The proposed VA-d-μ-SPE method coupled to HPLC is superior to the others in term of high analytical performance, short analysis time, and being environmentally friendly since it requires just a low cost of sorbent. The sensitivity of the proposed method in term of LOD is almost comparable to that obtained from other microextraction methods. The presented method achieves low LODs, which are below the MRLs of neonicotinoid insecticide residues in agricultural products. 

## 3. Experimental

### 3.1. Chemicals and Reagents

All reagents were analytical grade unless otherwise specified. Analytical standard grade neonicotinoid insecticides including thiametoxam, clothianidin, imidacloprid, and acetamiprid were obtained from Dr. Ehren-Storfer (Storfer, Augsburg, Germany), and thiacloprid was obtained from Sigma-Aldrich (Schnelldorf, Germany). The stock solution containing 1000 mg L^−1^ of each insecticide was prepared in methanol (MeOH) and the working solution were acquired by diluting the stock solution with water. HPLC grade of acetonitrile and methanol were obtained from Merck (Darmstadt, Germany). Sodium chloride (NaCl), anhydrous sodium carbonate (anh. Na_2_CO_3_), and anhydrous sodium sulphate (anh. Na_2_SO_4_) were obtained from Ajax Finechem (New Zealand). Sodium acetate (CH_3_COONa) was purchased from Carlo Erba (France). Montmorillonite (MMT) Cloisite 10A clay was obtained from Rheologie additive (Germany). All solutions were prepared in deionized water obtained from RiOs™ Type I Simplicity 185 (Millipore Waters, Milford, MA, USA) with a resistivity of 18.2 MΩ·cm.

### 3.2. Apparatus

A Waters 1525 Binary HPLC pump (Millipore Waters, Milford, MA, USA) equipped with a diode array detector (DAD) was used for chromatographic separation. A Rheodyne injector with 20 µL injection loop was used. For the system control and data processing, Empower 3 software was used. Due to their polarity of the target analytes, a reverse-phase C18 column was chosen. A LiChrospher^®^ 100 RP-18 end capped (4.6 × 150 mm, 5.0 µm) analytical column (Merck, Darmstadt, Germany) maintained at ambient temperature was used. Chromatographic separation of the target residues was performed using isocratic elution with 25% (*v*/*v*) acetonitrile in water as the mobile phase at a flow rate of 1.0 mL min^−1^ and detection wavelength was set at 254 nm. It was found that the five studied neonicotinoid insecticides were separated within 13 min with the elution order of thiametoxam (t_R_ = 4.38 min), clothianidin (t_R_ = 5.77 min), imidacloprid (t_R_ = 6.46 min), acetamiprid (t_R_ = 7.78 min), and thiacloprid (t_R_ = 12.24 min).

### 3.3. VA-d-µ-SPE Procedure

**Figure 8:**
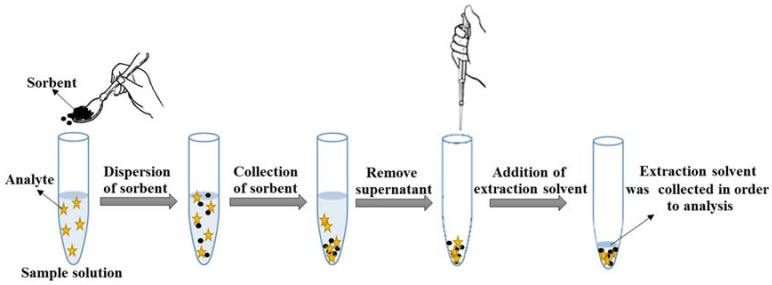
Schematic diagram of the proposed extraction method.

The determination of neonicotinoid insecticides was carried out by the VA-d-µ-SPE procedure using MMT Cloisite 10A clay sorbent followed by HPLC-PDA. Figure 8 shows schematic diagram of the proposed extraction method. For this method, in the VA-d-µ-SPE step, an aliquot of 13 mL of aqueous standard or sample solution was mixed with 0.03 g of Na_2_SO_4_ before being placed in a 15 mL centrifuge tube containing 30 mg of MMT Cloisite 10A clay sorbent. A suspension was formed and then placed the mixture in vortex agitation for 1 min and centrifuged at 3500 rpm for 10 min to enhance the sorption of the target analytes onto the sorbent. After that, the clear supernatant was removed by syringe. Then, the eluted target analytes which absorbed on the clay sorbent were extracted using 70% (*v*/*v*) acetonitrile. The mixture was vortexed for 2 min and centrifuged at 3500 rpm for 5 min. The clear supernatant was kept and filtered through a 0.45 μm nylon membrane filter before analysis by HPLC. 

### 3.4. Preparation of Samples

Three natural surface water samples were taken from different areas located near rice fields in Maha Sarakham (Northeastern of Thailand) province and were filtered through a Whatman No. 42 filter paper. Then, the filtrate was filtered through a 0.45 μm nylon membrane filter.

Fruit samples (watermelon, grape, and longan) were purchased from different markets in Maha Sarakham province. Before analysis, a 30.0 mL aliquot of fruit juice was centrifuged at 3500 rpm for 15 min and was filtered through Whatman No. 42 filter paper. Then, the solution was filtered through a 0.45 mm nylon membrane filter before extraction by the proposed method. Matrix-match calibration was prepared by the above-mentioned procedure and was followed by adding an appropriate concentration of standard solution before applying the extraction method. For accuracy evaluation, the studied samples were spiked with standard neonicotinoids into the homogenized samples prior to the extraction and preconcentration step.

## 4. Conclusions

In the present study, a simple vortex-assisted dispersive micro-solid phase extraction (VA-d-μ-SPE) method was proposed for preconcentration of neonicotinoid insecticides prior to analysis by HPLC. Montmorillonite was used as a solid sorbent for extraction of the target analytes. The method represented here has acceptable relative recoveries, good repeatability, and a wide linear range. When compared to other extraction methods for neonicotinoids analysis, this method reduces the exposure to toxic solvents used in the conventional extraction procedures, is environmentally friendly since it requires just a low cost of sorbent and has a much faster extraction time with high extraction efficiency. The method showed reliability with an appropriate analytical detection range for application in natural surface water and fruit juice samples. 

## Figures and Tables

**Table 1:** Analytical performance of the proposed method.

Insecticide	Linear Equation	Linearity (ng mL^−1^)	Coefficient of Determination (*R^2^*)	LOD (ng mL^−1^)	LOQ (ng mL^−1^)	Intra-Day (n = 3) %RSD		Inter-Days (*n* = 3 days × 3) %RSD		EF
						t_R_	Peak Area	t_R_	Peak Area	
**Thiamethoxam**	y = 1894030x + 256	0.5–1000	0.9951	0.009	0.031	0.46	2.75	0.21	4.58	73
**Clothianidin**	y = 3990993x − 3842	0.5–1000	0.9972	0.013	0.042	0.30	2.97	0.16	5.71	176
**Imidacloprid**	y = 1385642x – 6607	0.5–1000	0.9940	0.006	0.010	0.30	2.85	0.16	5.10	8
**Acetamiprid**	y = 945451x – 4414	0.5–1000	0.9963	0.005	0.008	0.28	3.24	0.44	4.99	8
**Thiacloprid**	y = 2249620x + 43266	0.5–1000	0.9980	0.065	0.263	0.23	3.26	0.14	7.17	16

LOD: limit of detection, LOQ: limit of quantitation, RSD: relative standard deviation, EF: enrichment factor.

**Table 2:** Analysis of neonicotinoid insecticides in real samples.

Sample	Market	Amount Found ± SD, μg mL^−1^ (*n* = 2)				
		Thiamethoxam	Clothianidin	Imidacloprid	Acetamiprid	Thiacloprid
Watermelon	Local Market	0.03 ± 0.007	0.05 ± 0.003	-	-	0.007 ± 0.01
	Super Market	0.11 ± 0.08	0.01 ± 0.007	0.07 ± 0.1	0.27 ± 0.1	0.005 ± 0.01
Grape	Local Market	0.04 ± 0.01	0.04 ± 0.002	-	0.06 ± 0.03	0.01 ± 0.01
	Super Market	0.26 ± 0.02	0.03 ± 0.003	-	-	-
Longan	Local Market	-	-	-	-	-
	Super Market	-	-	-	-	-
Water sample	Water I	-	-	-	-	-
	Water II	-	-	-	-	-
	Water III	-	-	-	-	-

- : Not detected.

**Table 3:** Comparison of the proposed VA-d-µ-SPE method with other sample preparation methods for the determination of neonicotinoids.

Method	Analytes	Sample	Analytical Technique	Linearity	LOD	Recovery (%)	Reference
**DSPE-DLLME**	Nitenpyram, Dinotefuran, Clothianidin, Thiamethoxam, Acetamiprid, Imidacloprid, Thiacloprid	Grain	HPLC-DAD	0.02–4.5 μg mL^−1^	0.002–0.005 mg kg^−1^	76–123	[14]
**VSLLME-SFO**	Acetamiprid, Clotianidin, Nitenpyram, Imidacloprid, Thiamathoxam	Water and fruit juice	HPLC-DAD	0.0005-5 µg mL^−1^	0.1–0.5 μg L^−1^	85–105	[12]
**SPE**	Acetamiprid, Imidacloprid, Thiacloprid, Thiamethoxam	Drinking water	LC-ESI-MS	0–1 mg L^−1^	0.01 µg L^−1^	95–104	[38]
**DLLME**	Acetamiprid, Clothianidin, Thiamethoxam, Imidacloprid, Dinotefuran, Thiacloprid, Nitenpyram	Honey	LC-MS/MS	1.5–100.0 µg kg^−1^	0.5–1.0 μg kg^−1^	74.3–113.9	[39]
**SPE-DLLME**	Thiamethoxam, Clothianidin, Imidacloprid, Acetamiprid, Thiacloprid	Honey	LC-APCI-IT-MS/MS	0.1–7500 ng g^−1^	0.2–1.0 ng g^−1^	90–104	[40]
**DLLME**	Thiacloprid, Acetamiprid, Imidaclothiz, Imidacloprid	Cucumber	MEKC	2.7–200 ng g^−1^	0.8–1.2 ng g^−1^	79.7–98	[4]
**VA-D-µ-SPE**	Imidacloprid, Acetamiprid, Clothianidin, Thiacloprid, Thiamethoxam	Fruit juice and natural surface water	HPLC-DAD	0.5–1000 ng mL^−1^	0.005–0.065 ng mL^−1^	8–176	This study
